# Ball Milling Promoted *N*-Heterocycles Synthesis

**DOI:** 10.3390/molecules23061348

**Published:** 2018-06-04

**Authors:** Taghreed H. El-Sayed, Asmaa Aboelnaga, Mohamed A. El-Atawy, Mohamed Hagar

**Affiliations:** 1 Chemistry Department, Faculty of Science, Taibah University, Yanbu 46423 Saudi Arabia; taghreed.hussien@yahoo.com (T.H.E.-S.); asmaa_aboelnaga77@yahoo.com (A.A.); mohamed.elatawi@alexu.edu.eg (M.A.E.-A.); 2 Chemistry Department, Faculty of Women for Arts, Science and Education, Ain Shams University, Heliopolis, Cairo 11757, Egypt; 3 Chemistry Department, Faculty of Science, Alexandria University, P.O. 426 Ibrahemia, Alexandria 21321, Egypt

**Keywords:** ball milling, green synthesis, mechanochemistry, *N*-heterocycles

## Abstract

In the last years, numerous protocols have been published using ball milling for organic synthesis. Compared to other methods such as microwave or ultrasound irradiation and ionic liquids, ball mill chemistry is an economical, and ecofriendly method in organic synthesis that is rather underrepresented in the knowledge of organic chemists. The aim of this review is to explore the advantages of the application of ball milling in synthesis of *N*-heterocyclic compounds.

## 1. Introduction

The past decade has witnessed a sustainable and ever increasing interest in the reactivity of *N*-heterocycles due to their importance in Nature, medicinal chemistry, living matter, drug design, advanced materials, and natural product synthesis [1]. They constitute an important an important class of compounds found in many natural products [2] with anticancer [3], cytotoxic [4], anti-HIV [5], anti-malarial [6], anti-inflammatory [7], antimicrobial [8], anti-hyperglycemic and anti-dyslipidemic [9] activity, in addition to anti-neurodegenerative disorder drugs targeting Alzheimer’s, Parkinson disease, Huntington’s disease [10], and many more [11,12].

Many reviews involving the synthesis of *N*-heterocycles have been published, including cascade synthetic reactions [13], the synthesis of six membered rings [14], the synthesis of spiro hetrocycles [15], the microwave assisted synthesis of five-membered azaheterocyclic systems [16], synthesis of small *N*-hetrocycles [17] and metalation [18].

Ball milling is a mechanical method broadly used to granulate minerals into very fine particles and the preparation or alteration of inorganic solids, although its use in organic synthesis is relatively uncommon [19,20,21,22].

**Figure 1:**
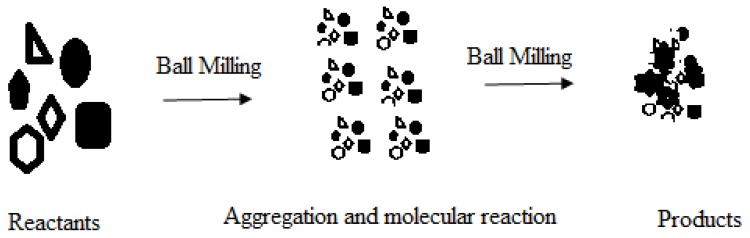
Ball milling process.

The term mechanochemistry has been introduced in periodicals recently. According to IUPAC a mechanochemical reaction is defined as “a chemical reaction that is induced by the direct absorption of mechanical energy” [23]. However, the area is furthermore divided into: (i) mechanical activation of solids; (ii) mechanical alloying and (iii) the reactive milling of solids [24,25,26,27] (Figure 1). Very recently, ball milling has been used in synthesis of organic compounds. Several reviews describing the use of ball milling in the synthesis and reactions of organic compounds have been published [28,29,30,31,32]. 

Recently, ball milling reactions have not been limited to simple organic reactions like condensations but have become widely used in more complex reactions like:

- Metal-catalyzed organic reactions
- nucleophilic reactions
- cascade reactions
- Diels–Alder reactions
- Oxidation-reduction reactions
- Halogenation and aminohalogenation reactions
- Formation of calixarenes, rotaxanes and cage compounds
- Transformation of biologically active compounds
- Asymmetric synthesis

This review is dedicated to the utilization of the ball-milling technique in the synthesis of heterocycles.

## 2. Five Membered Rings 

### 2.1. Pyrrole Synthesis 

**Scheme 1:**
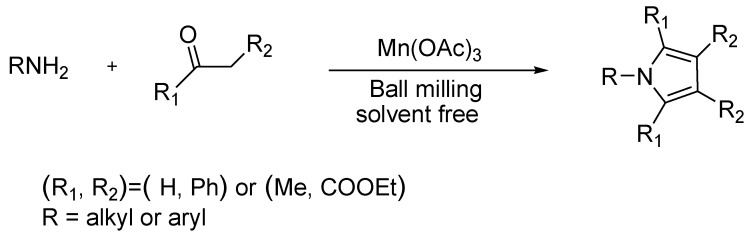
Synthesis of 2,5-dimethylpyrrole-3,4-dicarboxylates and 3,4-diphenylpyrroles.

Zeng et al. [33] developed a mechanochemical and solvent free synthesis for preparing a series of 2,5-dimethylpyrrole-3,4-dicarboxylates and 3,4-diphenylpyrroles in moderate to excellent yields from various amines and acetoacetate or 2-phenylacetaldehyde, respectively, in the presence of Mn(OAc)_3_ as mediator (Scheme 1).

**Scheme 2:**
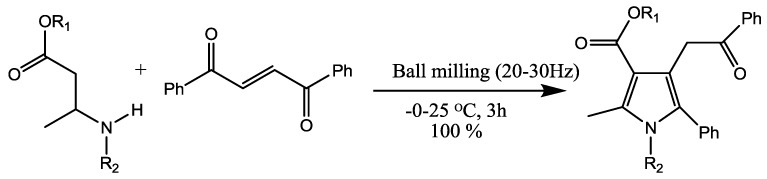
Reaction of trans-1,2 dibenzoylethene with primary, secondary enamine esters or enamine ketones.

Cascade mechanical milling reactions were reported for the first time by Kaupp et al. [34]. They investigated synthesis of pyrrole and indole products in quantitative yields by the reaction of trans-1,2 dibenzoylethene with primary, secondary enamine esters or enamine ketones in a ball mill (Scheme 2). The reactions took place through Michael addition of the enamine nitrogen followed by cyclization addition of the enamine double bond. The product is obtained by rearrangement to the enamine followed by elimination of water.

### 2.2. Indole Synthesis.

**Scheme 3:**
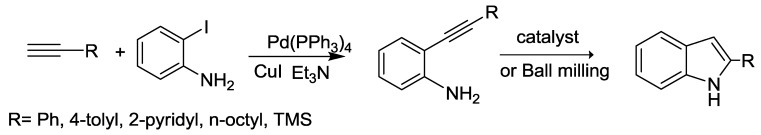
Sonogashira reaction for synthesis of indoles.

Zille et al. [35] reported a good Sonogashira reaction method under solvent free conditions involving the reaction of *o*-iodoaniline and terminal alkynes using ZnBr_2_ as catalyst to afford 2-alkynylanilines in a planetary ball mill at 800 rpm for 30 min (Scheme 3).

**Scheme 4:**
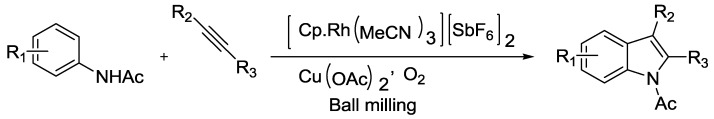
Oxidative cyclization of acetanilides and non-terminal alkynes.

Rhodium (III)-catalyzed oxidative cyclization of acetanilides and non-terminal alkynes using dioxygen as a oxidant in the absence of a solvent, under mechanochemical conditions afforded 10 differently substituted indole in moderate to good yields [36] (Scheme 4).

**Scheme 5:**
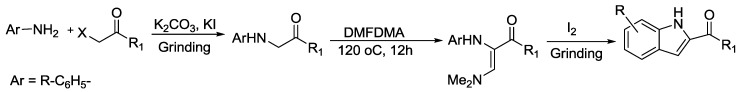
Cyclization of enaminone for synthesis of 2-carbonylindoles.

Under solvent-free and milling reaction conditions, 2-carbonylindoles were synthesized by cyclization of their corresponding enaminone using molecular iodine as a mediator for the annulation process. The enaminone precursors were prepared by mechanochemical reaction of aniline derivatives with alpha-haloketone to afford arylaminomethylenecarbonyl derivative then by subsequent thermal condensation with *N,N*′-dimethylformamide dimethyl acetal [37] (Scheme 5).

**Scheme 6:**
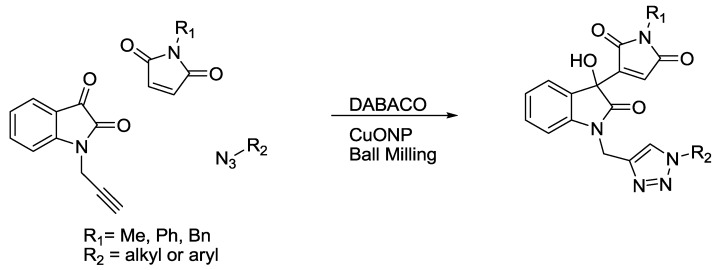
Synthesis of *N*-triazolylmethyloxindole.

Recently, Vadivelu et al. reported solvent-free ball milling of three components—malimide, *N*-propargyl isatin and an alkyl or aryl azide—along with DABCO and CuO nanoparticles as a recyclable heterogeneous catalyst to afford *N*-triazolylmethyloxindole [38] (Scheme 6).

### 2.3. Indeno[1,2-b]pyrrole Synthesis

**Scheme 7:**
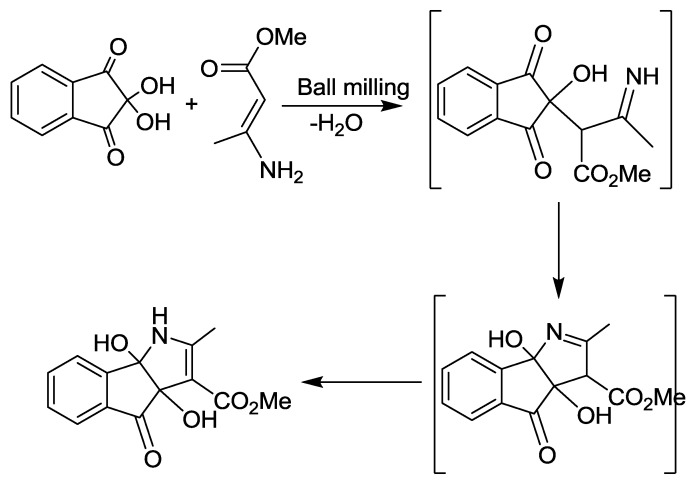
Synthesis of indeno[1,2-b]pyrrole.

Synthesis of *N*-heterocyclic compounds with α-hydroxyketone and N=O-semiaminal functionalities had been reported by Kaupp et al. [39]. The reaction proceeded via a three-cascade reactions vinylogous substitution, cyclization, and 1,3-hydrogen shift by reaction of ninhydrin and enamino ester (Scheme 7).

### 2.4. Pyrazole Synthesis

**Scheme 8:**
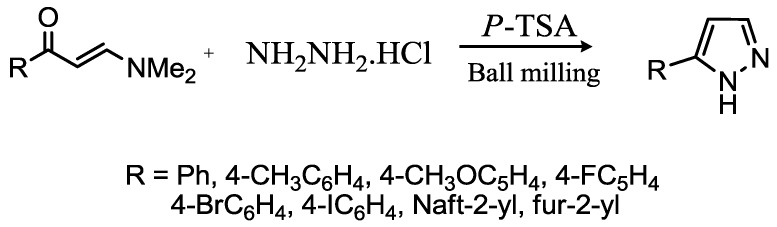
Synthesis of 1*H*-pyrazole derivatives.

Paveglio et al. [40] studied the mechanical parameters for the best conversion and selectivity for synthesis of 1*H*-pyrazole derivatives in a ball mill (Scheme 8.) The optimum conditions were 450 rpm, five balls (10 mm), and the use of 10% of *para*-toluenesulfonic acid (*p*-TSA) as catalyst for 3 min.

**Scheme 9:**
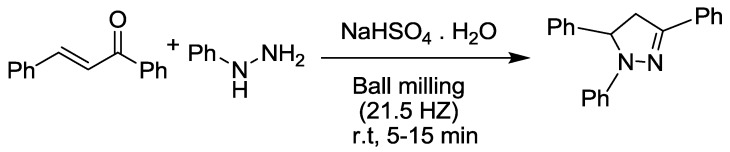
Synthesis of 1,3,5-triaryl-2-pyrazoline.

A solid-solid ball milling reaction of chalcone and phenylhydrazine catalyzed by NaHSO_4_·H_2_O (0.5 equivalents) was reported by Zhu and coworkers [41]. The reaction proceeded effectively using a high-speed ball mill at 1290 rpm to give 1,3,5-triaryl-2-pyrazoline in good yields (up to 93%) (Scheme 9). The reaction was extended by using thiosemicarbazides and aliphatic enones to give 2-pyrazoline derivatives [28]. 

**Scheme 10:**
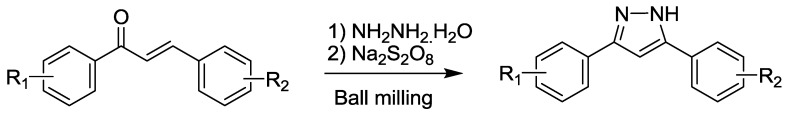
Synthesis of 3,5-diphenyl-1*H*-pyrazoles.

Ze et al. [42] developed a one-pot and solvent-free protocol for the synthesis in excellent yields of 3,5-diphenyl-1*H*-pyrazoles under mechanochemical ball-milling conditions using cheap sodium persulfate as the oxidant (Scheme 10) followed by a very simple work-up procedure.

**Scheme 11:**
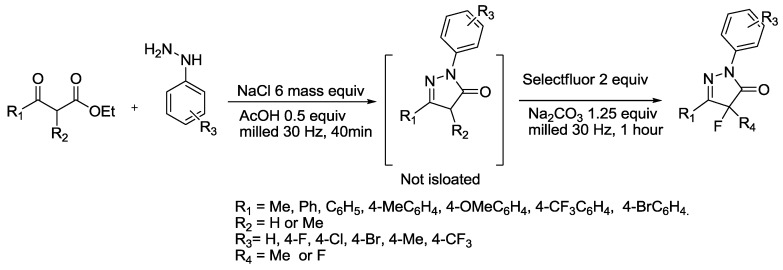
Condensation of a β-ketoester and phenylhydrazine.

Twelve diflourinated pyrazolones were synthesized via a solventless one-pot, two-step mechanochemical reaction. The first step is the condensation between a β-ketoester and phenylhydrazine to give the corresponding pyrazoline, which is flourinated in the next step to afford the fluorinated pyrazolones [43] (Scheme 11).

**Scheme 12:**
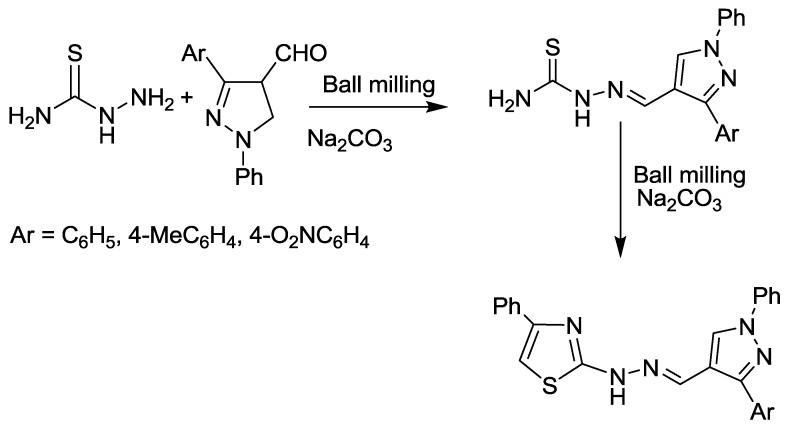
Synthesized of 2-(arylidenehydrazino)-4-phenylthiazoles.

Bondock and coworkers [44], synthesized a series of pyrazolylthiosemicarbazones by reaction of thiosemicarbazide and appropriate aldehydes using sodium carbonate and 1 h ball milling. The reaction of phenacyl bromide with pyrazolylthiosemicarbazones afforded the corresponding 2-(arylidenehydrazino)-4-phenylthiazoles in high yield (up to 98%) (Scheme 12).

### 2.5. Imidazole Synthesis

**Scheme 13:**
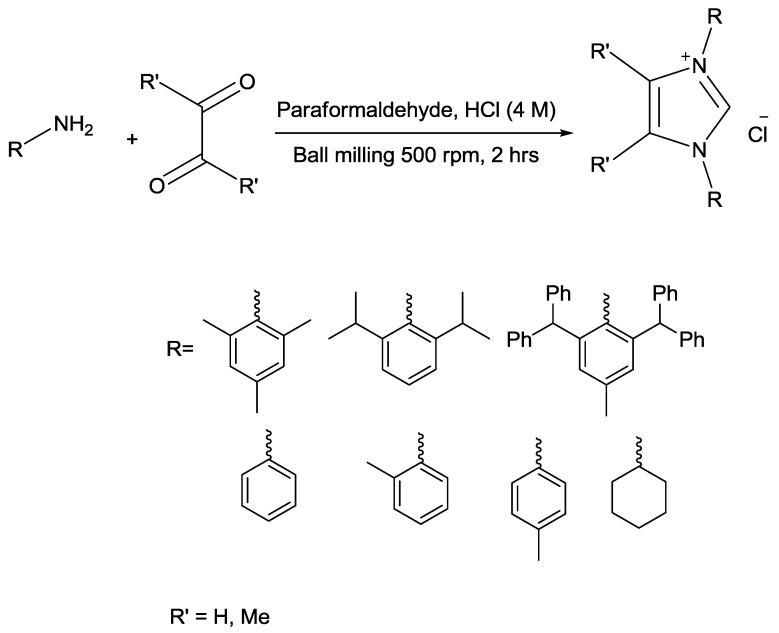
Synthesis of *N*-heterocyclic carbenes directly from anilines.

Lamaty et al. [45] investigated a solvent-free ball milling one-pot two-step synthesis of *N*-heterocyclic carbenes directly from anilines. This strategy allowed a significant improvement of the yields compared to conventional procedures. Synthesis of IPr^Me^·HCl of was selected as a model reaction. The optimum reaction conditions were 2:1 molar equivalents of 2,6-diisopropyl- phenylamine:2,3-butanedione at 500 rpm for two hours. Variable carbon sources were used (formaldehyde, chloromethylethylether, 1,3,5-trioxane and paraformaldehyde), and the best results were obtained with paraformaldehyde and HCl (4M) in dioxane as a solvent to afford the product in 49% yield over the two steps. Under the optimium conditions, the scope of the reaction was studied for many products (IPr·HCl, IMes·HCl, Io-Tol·HCl and ICy·HCl), and the reaction proceeded effectively to afford NCH in high yield (up to 100%) for all substrates except a highly hindered 2,6-diphenylmethyl-4-methylphenyl substrate (Scheme 13).

### 2.6. Benzimidazole Synthesis

**Scheme 14:**
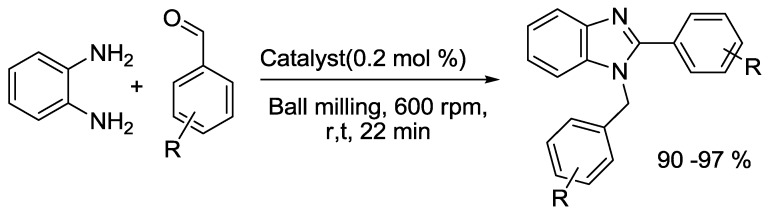
Synthesis of 1,2-disubstituted benzimidazoles.

Recyclable ionic liquid-coated ZnO-nanoparticles (ZnO-NPs, catalyst **5**) were employed as a catalyst in the green synthesis of 1,2-disubstituted benzimidazoles derivatives by a ball milling technique which produced high yields with high selectivity [46] (Scheme 14).

**Scheme 15:**
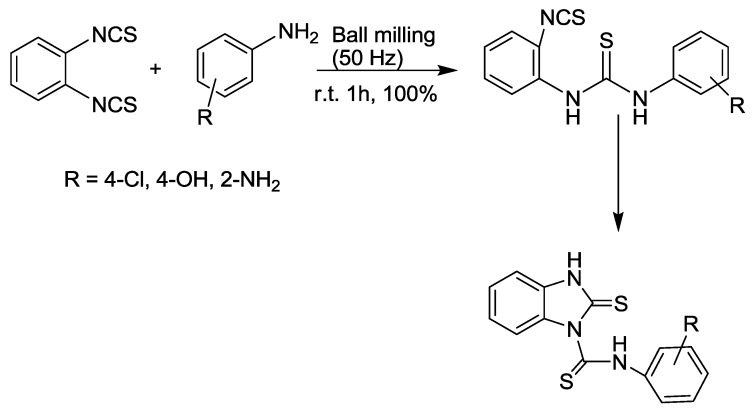
Synthesis of (anilino-thiocarbonyl)-benzimidazolidine-2-thiones.

At room temperature, 1 h ball milling afforded 100% yield of substituted (anilino-thiocarbonyl)-benzimidazolidine-2-thiones by reaction of aniline derivatives and *o*-phenylenediisothiocyanate (Scheme 15) [44].

**Scheme 16:**
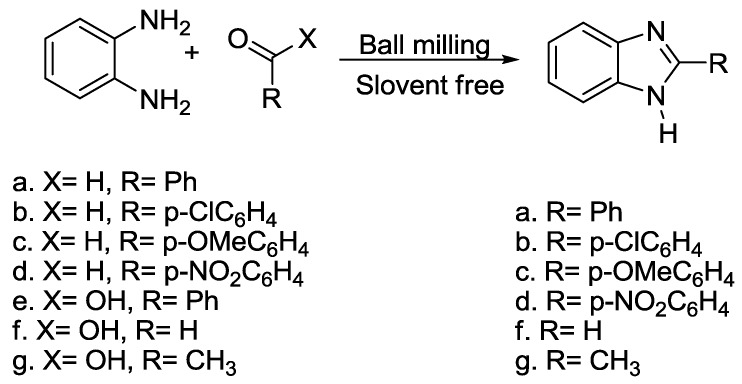
Synthesis of benzimidazoles from carboxylic acids or aldehydes.

**Scheme 17:**
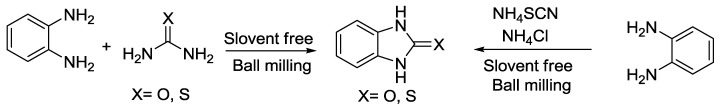
Synthesis of benzimidazol-2-ones or benzimidazol-2-thione.

**Scheme 18:**
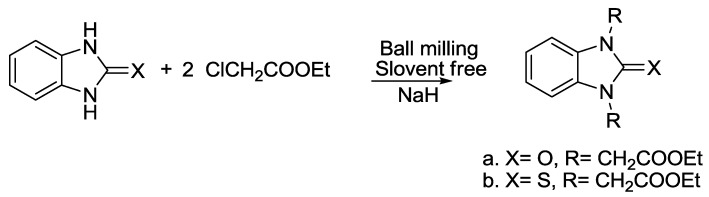
Alkylation of benzimidazolone or benzimidazolthione.

Recently, our research group [47] reported a high yielding ball milling synthetic method for a series of benzimidazol-2-ones or benzimidazol-2-thiones under solvent-free conditions by reaction of *o*-phenylenediamine and benzaldehydes or benzoic acids. Several reaction parameters were investigated such as milling ball weight, frequency and milling time. This method shows effectiveness for the reaction of different carboxylic acids, aldehydes, urea, ammonium thiocyanate or thiourea with o-phenylenediamine (Scheme 16 and Scheme 17). Moreover; alkylation of benzimidazolone or benzimidazolthione by ethyl chloroacetate was also studied (Scheme 18).

**Scheme 19:**
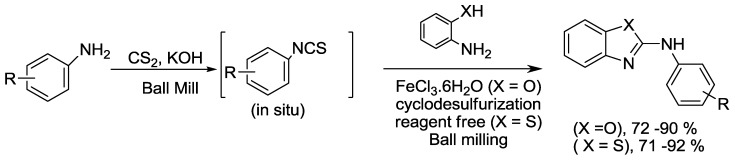
Synthesis of 2-anilinobenzoxazoles or 2-anilinobenzothiazoles.

Reaction of anilines, CS_2_, and 2-aminophenol or thiophenol under solvent-free ball milling conditions leads to a series of 2-anilinobenzoxazoles or thiazoles, respectively, in good to excellent yields (Scheme 19) [48].

### 2.7. Indeno[2,1-d]imidazole Synthesis

**Scheme 20:**
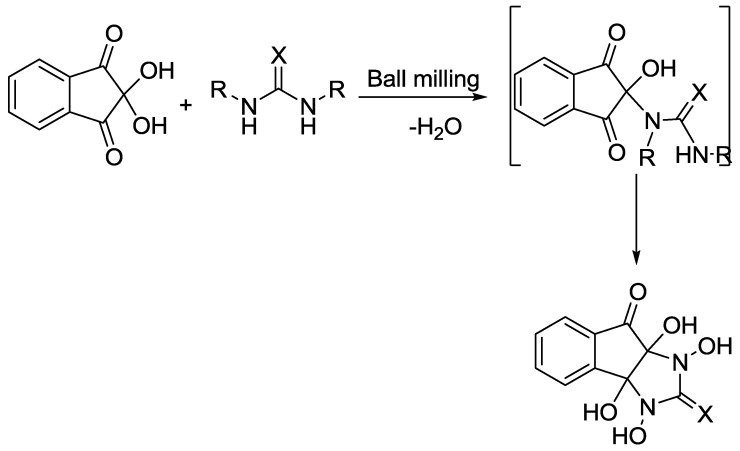
Reaction of ninhydrin with ureas/thioureas.

Kaupp et al. reported a good reaction of ninhydrin with ureas/thioureas to afford heterocyclic bis-semi acetals by substitution and addition cascade reactions under ball milling technique conditions [39] (Scheme 20).

### 2.8. Thiazole and Oxazole Synthesis

**Scheme 21:**
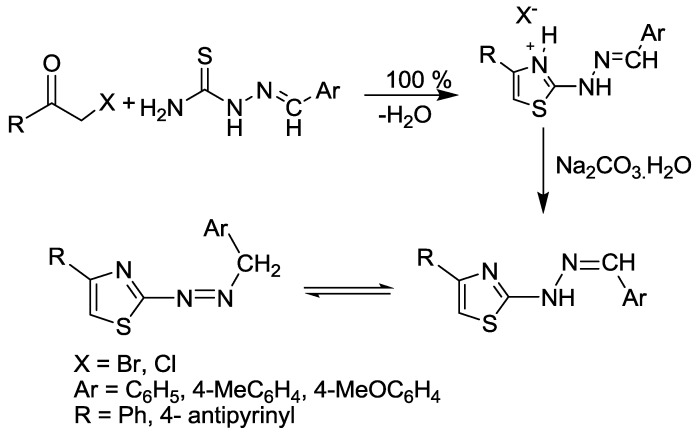
Synthesis of 4-substituted-2-(arylidenehydrazino)thiazoles.

Solvent and catalyst-free reactions of α-haloketones with thiosemicarbazones to give the corresponding 4-substituted 2-(arylidenehydrazino)thiazoles in a ball milling reactor were reported by Abdel-Latif and coworkers [49] (Scheme 21).

**Scheme 22:**
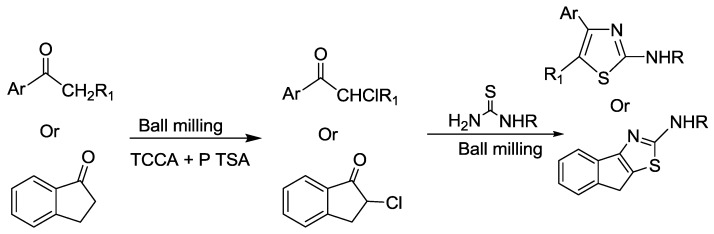
Synthesis of 2-hydrazinylthiazoles and 2-aminothiazoles.

Nagarajaiah et al. [50] reported an efficient chlorination method to give α-chloroketones by the reaction of ketones with trichloroisocyanuric acid in the presence of *p*-TSA under ball-milling conditions. Then these α-chloroketones reacted with thiosemicarbazides and thiourea to afford 2-hydrazinylthiazoles and 2-aminothiazoles, respectively, in good yields (Scheme 22).

**Scheme 23:**
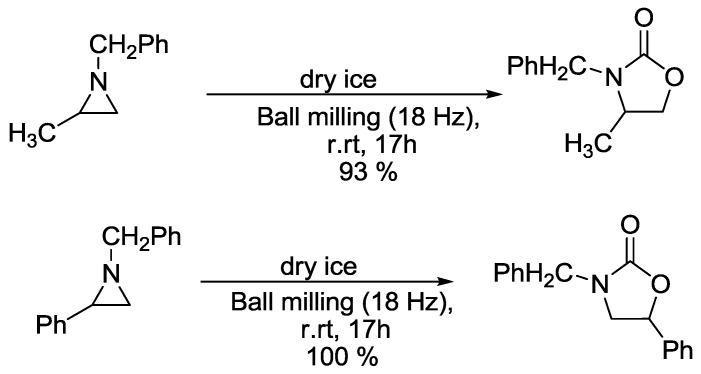
Conversion of aziridine into an oxazolidinone.

Phung et al. [49] showed the importance of the ball milling technique in dry ice for regioselective conversion of an unactivated 2-aryl aziridine or 2-alkyl into an oxazolidinone (Scheme 23).

### 2.9. Triazole Synthesis

**Scheme 24:**
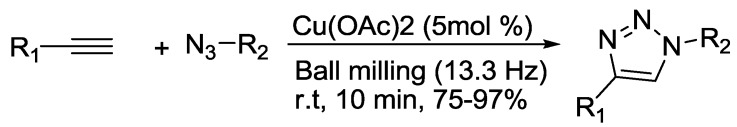
1,3-Dipolar cycloaddition of alkynes with decylazide.

A series of 1,4-substituted-1*H*-1,2,3-triazoles were synthesized in high yields (up to 99%) by 1,3-dipolar cycloaddition of alkynes with decylazide and a catalytic amount of Cu(OAc)_2_ using a ball-mill at 800 rpm rotation speed (13.3 Hz) for 10 min [51] (Scheme 24). 

**Scheme 25:**
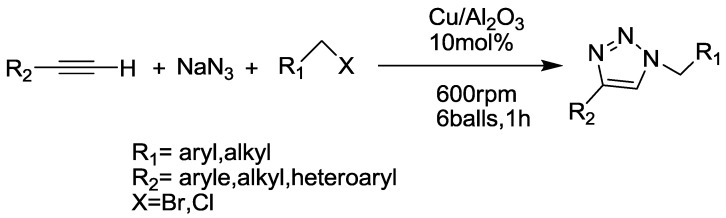
Coupling of terminal alkynes, alkyl halides or aryl boronic acids and sodium azide.

Under mechanical milling, synthesis of 1,2,3-triazole derivatives occurred by a coupling of terminal alkynes, alkyl halides or aryl boronic acids and sodium azide over copper(II) sulfate supported on alumina (Cu/Al_2_O_3_) in the absence of any solvent (Scheme 25) [52]. 

**Scheme 26:**
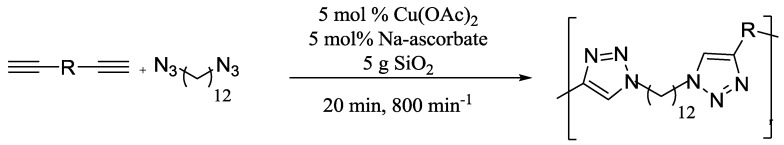
Polymerization of 1,12-diazidododecane and bisethynyl.

Thorwirth and coworkers [51] have reported polymerization of 1,12-diazidododecane and bisethynyl compounds in a ball mill without destroying the polymer backbone (Scheme 26).

**Scheme 27:**
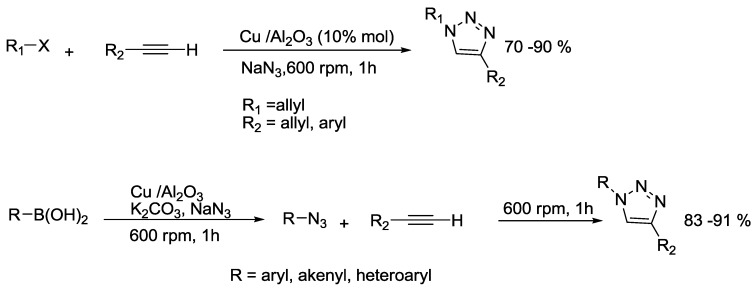
Synthesis of 1,4-disubstituted-1,2,3-triazoles from aryl boronic acid.

Ranu et al. [52] reported Cu/Al_2_O_3_ as a catalyst for the synthesis of 1,4-disubstituted-1,2,3-triazoles by the reaction of terminal alkynes, alkyl halide/aryl boronic acid and sodium azide under solvent free and ball milling conditions. This method averts the use of hazardous organo- azides to afford arylalkyl- and arylaryl-substituted 1,2,3-triazoles in excellent yield (Scheme 27). 

## 3. Six Membered Rings

### 3.1. Pyridine Synthesis 

**Scheme 28:**
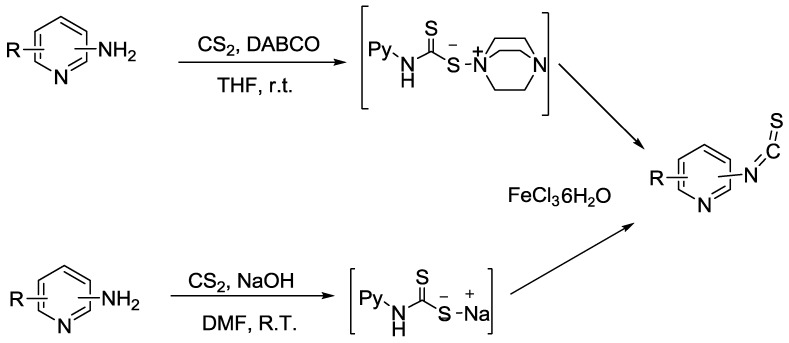
Synthesis of pyridyl isothiocyanates.

Zhang et al. [53] reported an effective method for the synthesis of pyridyl isothiocyanates (ITCs) from the corresponding amines, where aqueous iron(III) chloride promotes desulfurization of a dithiocarbamate salt that is generated in situ from the amine and carbon disulfide in the presence of DABCO or sodium hydride under ball-milling conditions ( Scheme 28). Use of this protocol gives good yields. 

### 3.2. Quinoline Synthesis

**Scheme 29:**
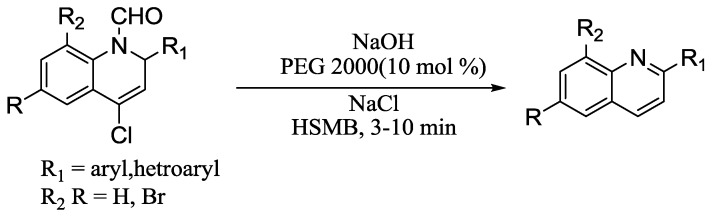
Deformylation of *N*-formyldihydroquinoline.

Yu et al. [54] reported a high yield (up to 99%) synthetic method for quinoline derivatives by the reaction of *N*-formyldihydroquinoline on a solid base such as sodium hydroxide (NaOH) under high-speed ball milling conditions with a catalytic amount of polyethylene glycol 2000 (PEG 2000) as catalyst (Scheme 29).

**Scheme 30:**
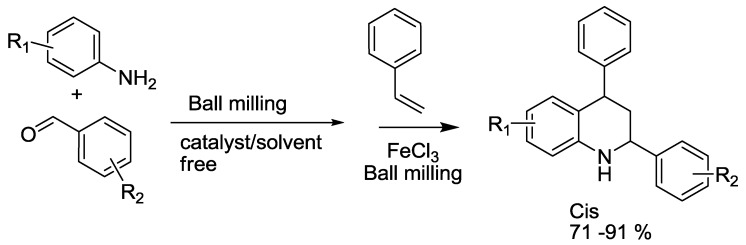
Diels–Alder synthesis of *cis*-2,4-diphenyltetrahydroquinolines.

Under solvent-free high-speed ball milling styrene and *N*-aryl aldimines generated in situ are used for the synthesis of *cis*-2,4-diphenyltetrahydroquinolines in good yield via Diels–Alder cycloaddition reactions in presence of FeCl_3_ (Scheme 30). This method is a very efficient and green alternative to conventional methods for synthesis for these types of heterocyclic skeletons. The advantages of this method are a short reaction time, easy availability of the required reagents, solvent free conditions and a nontoxic catalyst [53].

### 3.3. Imidazo[1,2-a]pyridine Synthesis

**Scheme 31:**
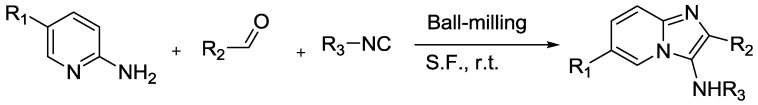
Ugi-multi-component reaction.

One pot Ugi-multicomponent reactions between 2-amioazines, aldehydes and isonitriles were conducted under solvent-free mechanochemical ball-milling conditions to afford 3-aminoimidazo[1,2-a]pyridine derivatives in good to excellent yields at room temperature [55]. The reaction has been shown to display good functional group tolerance (Scheme 31).

**Scheme 32:**
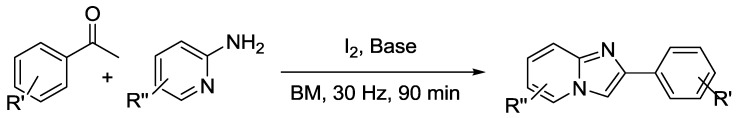
Synthesis of 2,3-substituted imidazo[1,2-a]pyridines.

A series of 2,3-substituted imidazo[1,2-a]pyridines were obtained by reaction of 2-amino- pyridines with methyl ketones or 1,3-dicarbonyl compounds by I_2_-enhanced condensation/ cyclization via ball milling under solvent-free conditions. This method gives good functional group and broad molecular diversity with good product yields [56] (Scheme 32).

### 3.4. Chromeno[3,4-b]pyridine Synthesis

**Scheme 33:**
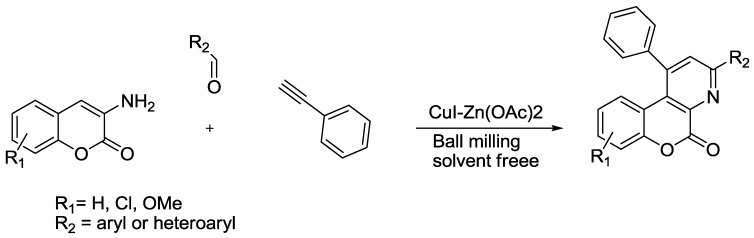
Synthesis of pyridocoumarins.

Recently, Kausar et al. described a synthesis of sixteen different pyridocoumarins in excellent yield by solvent free ball milling of 3-aminocoumarin, aldehydes and phenylacetylene along with CuI-Zn(OAc)_2_ as a catalyst for activation and functionalization of C(sp^2^)-H of 3-aminocoumarin [57] (Scheme 33).

### 3.5. Pyrimidine Synthesis

**Scheme 34:**
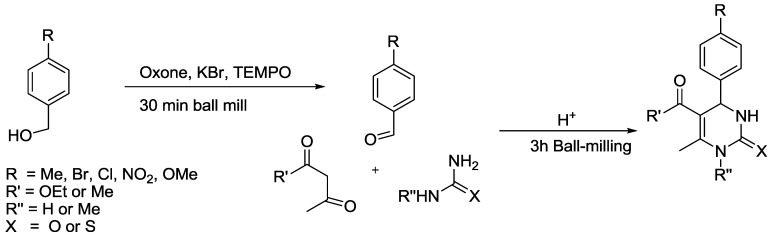
Multicomponent Biginelli reaction.

Under mechanochemical solvent-free conditions, a multicomponent Biginelli reaction was reported to give dihydropyrimidones [58]. The starting aldehydes were prepared within the same reaction pot by Br+ catalyzed oxidation of their corresponding primary alcohols which results in formation of byproducts. The acid was used as catalyst in the cascade transformation leading to dihydropyrimidones (Scheme 34).

**Scheme 35:**
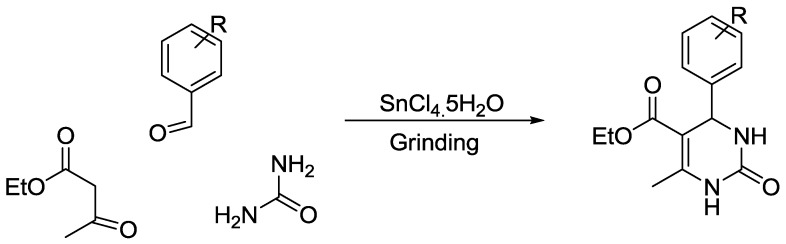
Synthesis of dihydropyrimidones by Biginelli reaction.

On the other hands, Sachdeva et al. reported the formation of dihydropyrimidones using a mechanochemical Biginelli reaction in presence of SnCl_4_·5H_2_O as a catalyst instead of the free acid [59] (Scheme 35).

**Scheme 36:**
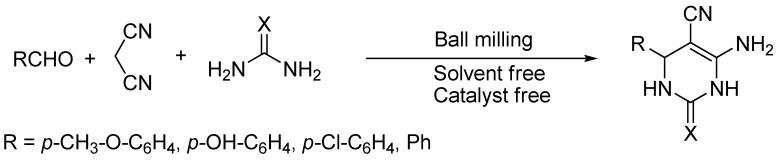
Synthesis of 2-thioxo or 2-oxo-1,2,3,4-tetrahydropyrimidine-5-carbonitriles.

Ould et al. [28] showed that the condensation reaction of an equimolar amount of an aldehyde, malononitrile and thiourea/urea by ball milling in 40 min gives 2-thioxo or 2-oxo-1,2,3,4-tetrahydropyrimidine-5-carbonitrile derivatives (Scheme 36). The reactions proceed effectively without the aid of any catalyst or solvent to give the products in excellent yields (up to 98%).

### 3.6. Pyrano[2,3-d]pyrimidine Synthesis

**Scheme 37:**
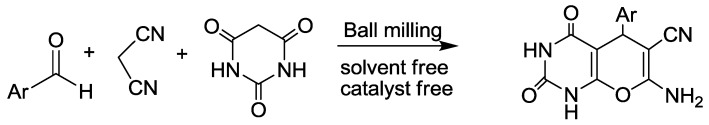
Synthesize pyrano[2,3-d]pyrimidine-2,4(1*H*,3*H*)-diones.

Mashkouri et al. [22] used aromatic aldehydes, malononitrile, and barbituric acid (Scheme 37) to synthesize pyrano[2,3-d]pyrimidine-2,4(1*H*,3*H*)-diones in good yield (up to 94%) under a ball milling technique in circulating warm water to heat the reaction for 55 min milling.

### 3.7. Diazine and Diazepine Synthesis

**Scheme 38:**
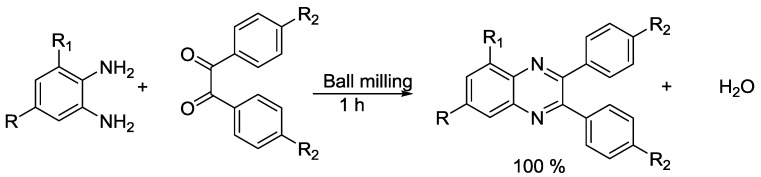
Condensation of *o*-phenylenediamines and benzil to quinoxaline.

Kaupp et al. [39] studied the condensation of *o*-phenylene diamines with various 1,2-dicarbonyl compounds under ball milling conditions to afford a series of heterocycles. The condensation reaction of substituted *o*-phenylenediamines and benzil gave quinoxaline derivatives within 1 h (Scheme 38). Moreover, was benzo[a]phenazin-5-ol obtained by a condensation reaction between 2-hydroxy1,4-naphthoquinone and *o*-phenylenediamine within 15 min at 70 °C.

**Scheme 39:**
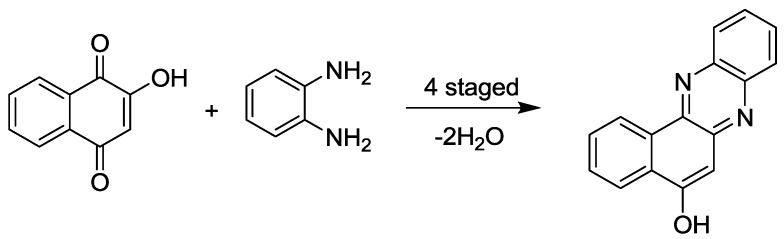
Cascade synthesis of benzo[a]phenazin-5-ols.

Substituted benzo[a]phenazin-5-ols were produced in 100% yield by milling of 2-hydroxy- 1,4-naphthoquinone and *o*-phenylenediamines for 15 min via a four cascade reaction (two additions two and eliminations, Scheme 39). 

**Scheme 40:**
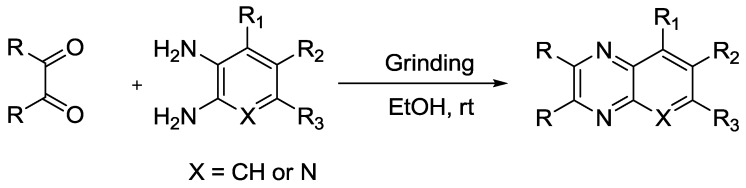
Synthesis of quinoxalines or pyrido[2,3-b]pyrazines.

Ball milling conditions were successfully was used in the condensation reactions between *o-*diaminoarenes with 1,2-dicarbonyl compounds to afford a variety of differently substituted quinoxalines or pyrido[2,3-b]pyrazines [60,61] (Scheme 40).

**Scheme 41:**
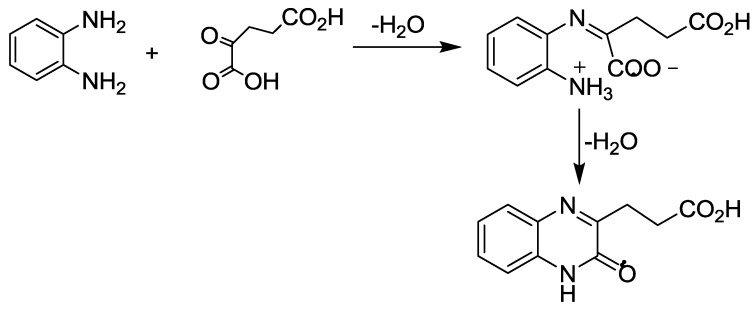
Synthesis of 3-oxo-3,4-dihydroquinoxaline.

**Scheme 42:**
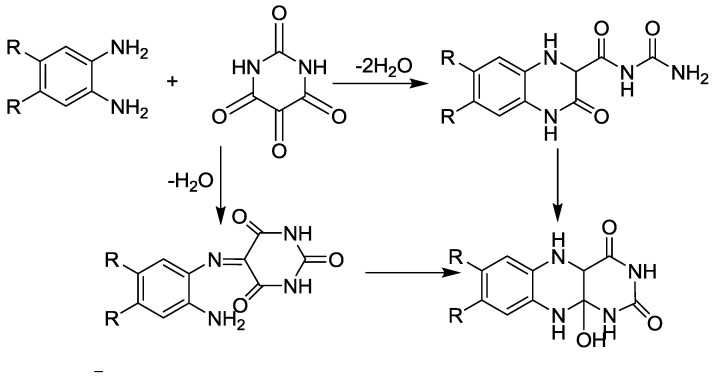
Synthesis of 3-oxo-3,4-dihydroquinoxaline-2-carbonyl-ureas.

3-Oxo-3,4-dihydroquinoxaline was produced in 90% yield by milling of 2-oxoglutaric acid and *o*-phenylenediamine for 10 min (Scheme 41). Four-cascade reactions consisting of substitution, elimination, cyclization and ring opening of *o*-phenylenediamines with alloxane hydrate and 3-oxo-3,4-dihydroquinoxaline-2-carbonylureas produced 3-oxo-3,4-dihydroquinoxaline-2-carbonyl- ureas [39] (Scheme 42).

**Scheme 43:**
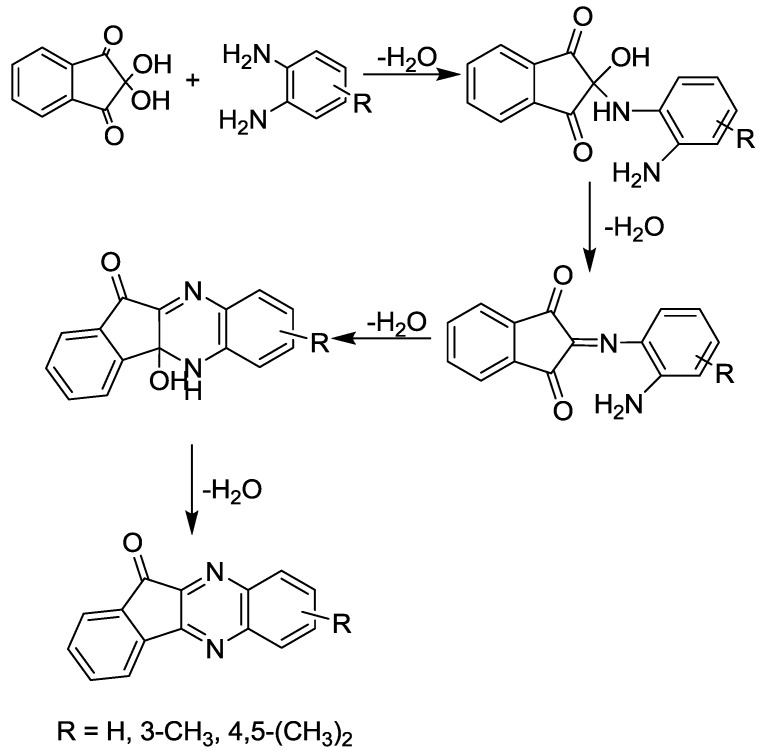
Synthesis of indenoquinoxaline ketones.

Kaupp et al. [62] described cascade mechanochemical reactions of solid-state ninhydrin and *o*-phenylenediamines, *o*-mercaptoaniline, urea/thiourea and methyl 3-aminocrotonate in a ball mill at 20–25 Hz to give indenoquinoxaline ketones (Scheme 43). 

**Scheme 44:**
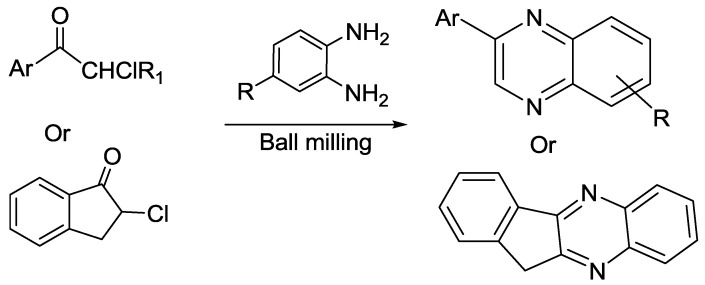
Reaction of α-chloroketones with *o*-phenylenediamine to quinoxalines.

Nagarajaiah et al. [50] reported an efficient ball-milling reaction of α-chloroketones with *o*-phenylenediamine to give quinoxalines (Scheme 44).

**Scheme 45:**
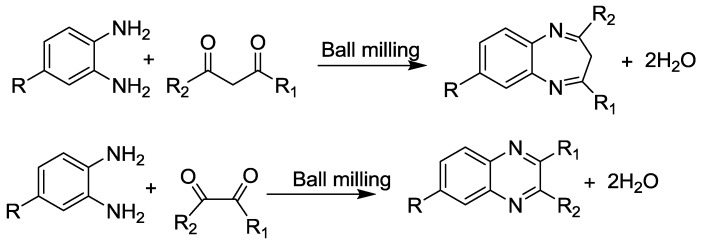
Synthesis of dibenzophenazines and dibenzopyridoquinoxaline.

Carlier et al. [63] described catalyst- and solvent-free mechanochemical reactions of diamines and 1,2- or 1,3-dicarbonyls to give dibenzophenazines and dibenzopyridoquinoxaline derivatives, respectively, in good yield (Scheme 45).

**Scheme 46:**
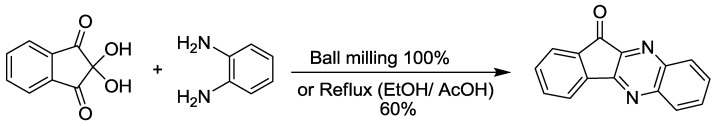
Reaction of ninhydrin with *o*-phenylenediamine.

Etman, et al. [64] reported an efficient reaction without the aid of any catalyst or solvent, of ninhydrin with *o*-phenylenediamine under ball milling conditions (Scheme 46). Using the conventional method gave only 60% yields of the same product by heating of ninhydrin with *o*-phenylenediamine in EtOH/AcOH (7:3).

### 3.8. Thiazine Synthesis

**Scheme 47:**
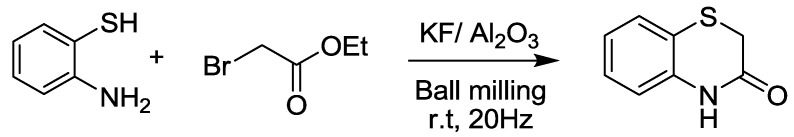
Synthesis of benzothiazinone.

Sharifi et al. [65] reported that the use of KF–Al_2_O_3_ solid support in a solvent-free ball milling procedure involving the reaction of 2-aminothiophenols with 2-bromoalkanoates (Scheme 47) led to a green and efficient synthesis of a series of benzothiazinone in excellent yield. 

**Scheme 48:**
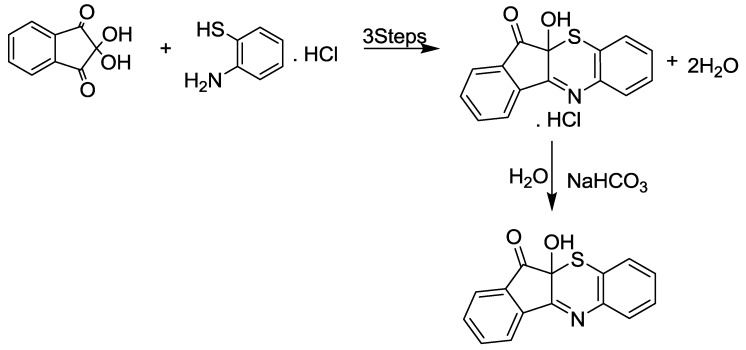
Synthesis of hydroxyindeno[2,1-b]benzo[1,4]thiazine.

Moreover, hydroxyindeno[2,1-b]benzo[1,4]thiazine was produced by the reaction of ninhydrin with *o*-mercaptoaniline hydrochloride in a in three-reaction cascade (substitution, cyclization and elimination, Scheme 48).

### 3.9. Azaborinine Synthesis

**Scheme 49:**
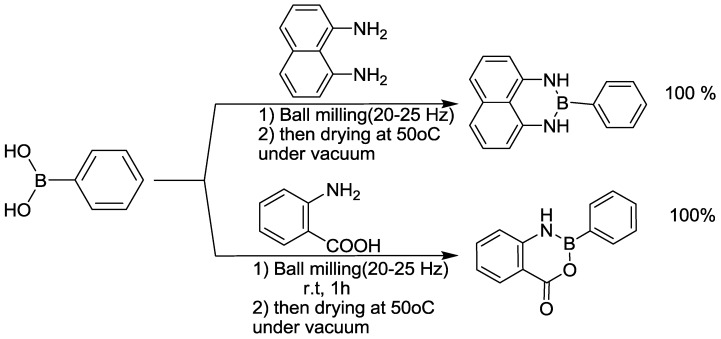
Synthesis of azaborinines.

The six membered heterocyclic diazaborinine and O, B, N six-membered heteroborinone could be obtained by mixing of 1,8-diaminonaphthalene or anthranilic acid and phenylboronic acid in a ball mill without solvent for 1 h followed by heating in a vacuum [30] (Scheme 49).

## 4. Higher Membered Heterocycles

**Scheme 50:**
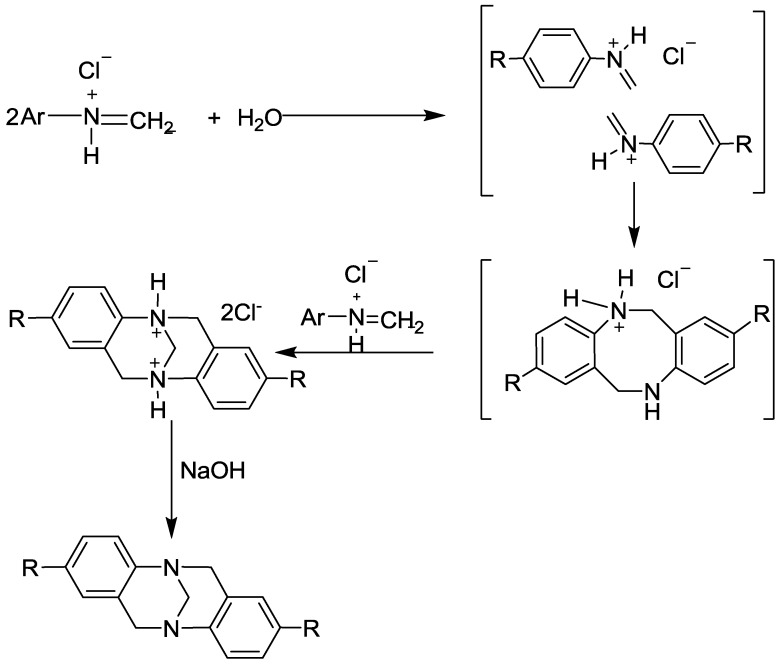
Synthesis of 1,5-diazocine.

Kaupp et al. [66] studied the transformation of *N*-arylmethyleneiminium salts to Tröger’s bases in the presence of water vapor or MgSO_4_·7H_2_O. after milling for 5–10 min. The products were formed via a three-reaction cascade involving a double arylaminomethylation and methylenation of the tetrahydro-1,5-diazocine intermediate (Scheme 50).

## 5. Conclusions

Herein, we have reviewed the use of mechanochemical technique for synthesis of variety of *N*-heterocyles. As discussed, the ball milling technique is becoming a more promising green tool for the synthesis of various *N*-heterocycles, including condensation reactions, multicomponent cascade reactions, metal catalyzed synthesis, etc.
